# Resolving relationships in the radiation of Australia’s largest pea clade (Fabaceae tribe Mirbelieae) with target-capture sequencing

**DOI:** 10.1093/aob/mcaf128

**Published:** 2025-06-17

**Authors:** James A R Clugston, Russell L Barrett, Daniel J Murphy, Matthew A M Renner, Peter H Weston, Lyn G Cook, Peter C Jobson, Brendan J Lepschi, Michael D Crisp

**Affiliations:** National Herbarium of New South Wales, Australian Botanic Garden, Mount Annan, NSW 2567, Australia; Montgomery Botanical Center, Coral Gables, FL 33156-4242, USA; Hawkesbury Institute for the Environment, Western Sydney University, Penrith, NSW 2751, Australia; National Herbarium of New South Wales, Australian Botanic Garden, Mount Annan, NSW 2567, Australia; Evolution and Ecology Research Centre, School of Biological, Earth, and Environmental Sciences, University of New South Wales Sydney, Kensington, NSW 2052, Australia; Royal Botanic Gardens Victoria, Melbourne, VIC 3004, Australia; National Herbarium of New South Wales, Australian Botanic Garden, Mount Annan, NSW 2567, Australia; National Herbarium of New South Wales, Australian Botanic Garden, Mount Annan, NSW 2567, Australia; School of Biological Sciences, The University of Queensland, Brisbane, QLD 4072, Australia; National Herbarium of New South Wales, Australian Botanic Garden, Mount Annan, NSW 2567, Australia; Centre for Australian National Biodiversity Research, Australian National Herbarium, a Joint Venture Between CSIRO and Parks Australia, Canberra, ACT 2601, Australia; School of Biological Sciences, The University of Queensland, Brisbane, QLD 4072, Australia; Research School of Biology, The Australian National University, Acton, ACT 2601, Australia

**Keywords:** Fabaceae, genomics, phylogenetics, Angiosperms353, Mirbelieae, target capture, next generation sequencing, phylogenomics, legumes, bush peas, *Pultenaea*, baits

## Abstract

**Background and Aims:**

Tribe Mirbelieae (Fabaceae) represents one of the great species radiations in Australian flora and the largest in the pea-flowered legumes. Traditional amplicon sequencing has failed to resolve relationships within this species-rich and morphologically diverse tribe.

**Methods:**

Target-capture sequencing was used to reconstruct relationships within the core Mirbelioid legumes, which represent a previously hypothesized rapid radiation that dates to the Oligocene and early Miocene epochs.

**Key Results:**

We recovered strongly supported deep nodes resolving relationships between all recognized genera and four novel clades based on 289 low-copy nuclear markers derived from the Angiosperms353 universal probe set. The taxonomically challenging genus *Pultenaea* was demonstrated to be polyphyletic. Minor changes are required in *Aotus*, *Callistachys*, *Mirbelia*, *Oxylobium*, *Phyllota* and *Urodon*.

**Conclusions:**

Phylogenomic data have robustly resolved relationships in a large legume clade where relationships were long feared irresolvable. This resolution enables the recognition of monophyletic genera within the tribe, with only minimal taxonomic rearrangements. Critically, a new circumscription of *Pultenaea* supported by both phylogenomic and morphological data has now been achieved.

## INTRODUCTION

Australia, an island continent, is home to ∼8 % of global flowering plant diversity (Govaerts *et al.*, 2021). Legume diversity is similarly high, and native species occupy almost all terrestrial biomes, representing almost 7 % of the Australian flora and ∼8 % of the global legume flora (Legume Phylogeny Working Group, 2021). Fabaceae tribe Mirbelieae (bush peas and their relatives), *sensu* Barrett *et al.* (2021), includes 24 currently accepted genera and 756 recognized species (almost 50 % of Australia’s native pea-flowered legumes), all but one of which are endemic to Australia. Within this tribe, the circumscription of genera in the *Pultenaea* Group *sensu* Crisp and Weston (1995; with 18 genera and >540 species recognized) has remained problematic. It has been proposed that difficulties in resolving deep nodes in the Mirbelieae might be attributable to the rapid radiation of this ecologically diverse and species-rich clade (Orthia *et al.*, 2005*a*, *b*; Barrett *et al.*, 2021). Relationships between core genera of Mirbelieae have remained unresolved, and several widely accepted genera are not monophyletic in their current circumscription, but a new classification of the tribe will require robust support for relationships (Orthia *et al.*, 2005*a*, *b*; Barrett *et al.*, 2021).

Given their high species diversity, the development of a well-resolved phylogenetic tree and robust classification of the tribe Mirbelieae is an important task. The tribe is of ecological and commercial importance through nitrogen fixation, provision of habitat for animals, agronomic potential through biocontrol (as the source of 1080 poison from species of *Gastrolobium* R.Br.; Peacock *et al.*, 2004), and being a major source of pollen and nectar for native bees and commercial honeybees (Mead *et al.*, 1985; Twigg *et al.*, 1996; Dear *et al.*, 2003; Robinson *et al.*, 2007; Ogilvie *et al.*, 2009; Sprent *et al.*, 2017; Kates *et al.*, 2024). Patterns of diversification within the tribe can also be expected to inform our understanding of the evolution of specific ecological adaptations in response to environmental change, such as drying climates (Byrne *et al.*, 2008; 2011), pollinator shifts (Crisp, 1994; Toon *et al.*, 2014) and biogeographical history of significant disjunctions in the Australian mesic flora (Byrne *et al.*, 2011; Weston and Jordon, 2017). Additionally, investigation of phylodiversity patterns can better guide conservation priorities by quantifying genetic diversity and determining conservation management units (Laity *et al.*, 2015; Broadhurst and Coates, 2017).

The first phylogenetic analyses of the Mirbelieae were based largely on morphological, anatomical and embryological characters and assumed that most of the genera then recognized were monophyletic (Crisp and Weston, 1987, 1995). However, the first phylogenetic tree for Mirbelieae based on molecular data was incongruent with both the earlier morphology-based trees (Crisp and Cook, 2003) and particularly challenged the monophyly of *Pultenaea* Sm., the largest genus in the Mirbelieae. These results also showed incongruence between plastid and nuclear markers, with a lack of resolution for most deep nodes in their analyses. Crisp and Cook (2003) drew attention to a large, strongly supported clade that showed weak internal resolution, which they called the ‘*Mirbelia* Group’ [but which Crisp and Weston (1995) had earlier given the informal name the ‘*Pultenaea* Group’]. Crisp and Cook (2003) suggested that perhaps all genera in this clade should be combined into one large genus, for which the name *Pultenaea* would have nomenclatural priority.

Orthia *et al.* (2005*a*) produced a relatively well-sampled phylogeny of the tribe, also based on *trn*L-F and ITS, but increased species sampling failed to increase the resolution of phylogenetic relationships. Orthia *et al.* (2005*b*) highlighted the incongruence between nuclear and plastid genetic markers as the most likely reason for this. Although many relationships were not strongly supported, Orthia *et al.* (2005*a*) did demonstrate that the largest genus in Mirbelieae, *Pultenaea* Sm., was not supported as monophyletic and that many taxa were geographically clustered, indicating that there is a link between genetic relationships and geography. Barrett *et al.* (2021) summarized the phylogeny of Mirbelieae using all available *trn*L-F data and hypothesized that hybridization, genome duplication, incomplete lineage sorting, and recent and rapid radiation might all be contributing to low phylogenetic resolution in the tribe. Renner *et al.* (2022) also found evidence for hybridization in *Pultenaea*, because interspecific hybridization was found between two species from eastern Australia, providing the first strong evidence of hybridization in the genus.

Representation of legumes in phylogenomic datasets is rapidly increasing, enabling the resolution of relationships that have confounded taxonomists for centuries (Vatanparast *et al.*, 2018; Koenen *et al.*, 2020*a*, *b*; Choi *et al.*, 2022). For example, recent studies have surveyed the entire Fabaceae for genes associated with nitrogen fixation (Folk *et al.*, 2021; Kates *et al.*, 2024) and resolved major clade relationships across the morphologically diverse family (Koenen *et al.*, 2020*a*; Aecyo *et al.*, 2021; Choi *et al.*, 2022; Ringelberg *et al.*, 2022). Next-generation sequencing has brought plant systematics into the genomic era (Godden *et al.*, 2012; McCormack *et al.*, 2013). Advances in DNA sequencing technologies have aided the development of targeted sequence capture through the enrichment of hundreds of informative markers throughout the genome that are cross-applicable through seed plants, offering a new dimension for understanding the phylogenetic relationship of species (Johnson *et al.*, 2019; Andermann *et al.*, 2020; Breinholt *et al.*, 2021).

The myBaits Angiosperms353 universal probe set has revolutionized our understanding of angiosperm evolution. It has provided unprecedented phylogenetic resolution for many plant groups, including Araliaceae, Cactaceae, Cleomaceae, Combretaceae, Cyperaceae, Gesneriaceae, Nepenthaceae, Gentianales, Myrtales and Poales (Larridon *et al.*, 2020; 2021*a*, *b*; Murphy *et al.*, 2020; Shee *et al.*, 2020; Antonelli *et al.*, 2021; Maurin *et al.*, 2021, 2023; Ogutcen *et al.*, 2021; Chincoya and Solórzano, 2022; Elliott *et al.*, 2024; GPWG III, 2024; Saunders *et al.*, 2024). The effectiveness of the Angiosperms353 baits to resolve even close evolutionary relationships has allowed it to become the standard as a universal probe set for angiosperm phylogenomics, and it has now been applied as a phylogenomic platform for exploring the Plant and Fungal Tree of Life (PAFTOL) and Genomics of Australian Plants (GAP) projects (Baker *et al.*, 2022; Zuntini *et al.*, 2024; Simpson *et al.*, 2025). Bioinformatic pipelines and workflows have been developed specifically to work with target-capture datasets (Johnson *et al.*, 2016; McLay *et al.*, 2021; Zhou *et al.*, 2022; Jackson *et al.*, 2023) to aid and improve their analysis, including the removal of paralogues and the detection of interspecific hybrids (Yang and Smith, 2014; Nauheimer *et al.*, 2021).

Here, we aim to create a robust phylogenetic framework for tribe Mirbelieae by applying next generation sequencing and target baits capture using the Angiosperms353 universal probe set. We also aim to test the monophyly of genera within the tribe, whose circumscriptions are, almost without exception, founded on morphological data. This will allow us to propose a durable generic classification for a group that has faced considerable taxonomic instability (Orthia *et al.*, 2005*b*). We anticipate the need to re-circumscribe some existing genera and the description of new genera to render the currently described genera monophyletic. We have already started this taxonomic process by revising the delimitation of *Pultenaea* as a clade of >150 species (many still unnamed; Renner *et al.*, 2022; Telford *et al.*, 2022; Barrett *et al.*, 2024*a*, *b*), all but one of which are restricted to eastern Australia. We have reinstated the genus *Euchilus* R.Br. from synonymy under *Pultenaea* and described three new genera of Western Australian species that were formerly included in *Pultenaea* (Barrett *et al.*, 2024*c*). Our new phylogenetic framework will also lay the foundation for a more detailed study into the origins and diversification processes within this uniquely Australian lineage and facilitate the delimitation of additional genera that remain non-monophyletic.

## MATERIALS AND METHODS

### Sample collection and preparation

We newly sampled 115 accessions of leaf material, mostly from wild populations across Australia, and either silica-dried or stored in CTAB gel (gel used for long term storage of DNA post-CTAB extraction). Fresh leaf samples were also collected from specimens in the living collections of the Australian Botanic Garden, Mount Annan, the Blue Mountains Botanic Garden, Mount Tomah and the Australian National Botanic Gardens, Canberra, with all samples supported by herbarium vouchers. Herbarium specimens collected within the last 30 years (where possible, but ≤120 years for some species) were sampled from the collections of BRI, CANB, MEL and NSW herbaria to fill any critical gaps owing to limitations on fieldwork during the coronavirus 2019 pandemic-related travel restrictions (see Supplementary Data Table S1).

### DNA extraction and library preparation

For samples from MEL, DNA extractions were based on a scaled-down version of the CTAB method used by McLay *et al.* (2022) for *Acacia pycnantha* Benth. For each sample, ∼100 mg of dried leaf material was placed in a 2 mL SafeLock Microcentrifuge tube (Eppendorf, Germany) along with a tungsten carbide bead and frozen in liquid nitrogen. The tissue was then disrupted using a TissueLyser II (Qiagen, Hilden, Germany). The resultant ground tissue was washed at least once with a sorbitol prewash before lysis for 2 h in a 2 % CTAB buffer with 1.4 m NaCl. After purification with chloroform:isoamyl alcohol, DNA in the resultant aqueous phase was precipitated as described by McLay *et al*. (2022) but at −20 °C for 1 h and centrifugation for 10 min at 21 300*g*. After two rounds of washing with 80 % ethanol, the dried DNA pellets were dissolved in 100 μL of 10 mm Tris–HCl pH 8. The concentration of each isolate was determined using a Qubit 3 Fluorometer (v.3.0 BR DNA assay; Invitrogen, Life Technologies, Carlsbad, CA, USA), and the purity of every second isolate was checked with a NanoDrop Lite Spectrophotometer (ThermoFisher Scientific Waltham, MA, USA). For samples from New South Wales, DNA extraction and library preparation for 96 samples was carried out by the Australian Genome Research Facility (AGRF). DNA extraction was carried out using a CTAB protocol (Porebski *et al.*, 1997) to maximize DNA output and yield, because many samples were stored within CTAB gel. Library preparation was completed using an NEB Next Ultra II FS kit (New England Biolabs, MA, USA) by AGRF. Target capture enrichment was completed using the myBaits Angiosperms353 v.1 universal phylogenomics solution for flowering plants developed by Johnson *et al.* (2019) and carried out by AGRF. Sequencing was then carried out using an Illumina NovaSeq 6000 sequencing platform for 150 bp paired-end reads in the single flow cell, with 300 cycles per sequencing run.

### Bioinformatics

#### Data assembly

Raw Illumina reads from Angiosperms353 sequencing for one species of each genus and outgroup taxa were sourced from the Genomics for Australian Plants project in partnership with Bioplatforms and Australian BioCommons Leadership Share (ABLeS) program Australia (Supplementary Data Table S1; https://australianbiocommons.github.io/ables/acknowledgements/; Simpson et al., 2025). Raw Illumina reads were visualized using Geneious Prime v.2022.0.12 (https://www.geneious.com), then checked for quality and consistency of base quality sores using FastQC v.0.11.9 (Andrews, 2010). Raw Illumina reads were filtered, with reads of <50 bp discarded using FastP v.0.23.1 (Chen *et al.*, 2018). Data were assembled using a modified version of HybPiper v.1.3.1 (Johnson *et al.*, 2016) named HybPiper-nf: containerized and pipelined using Singularity container (Kurtzer *et al.*, 2017) and a Nextflow pipeline (Jackson et al., 2023) using the standard settings without modification. Gene directories were then run within a modified version of the yang-and-smith pipeline (Yang and Smith, 2014), named paragon-nf (Jackson *et al.*, 2023), which contains additional options and bug fixes to detect potential paralogues created during the target enrichment process. The paragone-nf pipeline was used to remove these paralogues to align processed gene directories and produce three outputs using different algorithms: monophyletic outgroups (MO), rooted subtrees (RT) and maximum inclusion (MI), with each method having been described by Jackson *et al.* (2023). The MO output was used for our study, because the relationships between our outgroups were strongly supported by the much broader study of Zuntini *et al.* (2024).

#### Alignment and annotation

From the paragone-nf pipeline, the aligned gene directories from the MO algorithm were selected, because our outgroups were known to be monophyletic. Each gene alignment was visualized and checked using Geneious Prime 2022.0.2 (Biomatters Ltd, Auckland, New Zealand). Whole-gene alignments with significant missing data and evident alignment ambiguities were discarded. The remaining gene alignments were then concatenated using Geneious and exported in the NEXUS file format. Once alignments were concatenated, the matrix was checked for consistency of the alignment, then annotated by marker/gene within Geneious to export partitions for downstream phylogenetic analysis.

#### Phylogenetics

Maximum likelihood phylogenetic analysis was conducted using IQ-Tree v.2.3.6 (Minh *et al.*, 2020*a*) using a partition scheme determined by ModelFinder (Kalyaanamoorthy *et al.*, 2017) that was tested for each gene individually and rapid bootstrap analysis with 1000 bootstrap replicates and 1000 Single branch tests (-alrt). However, when a large marker set is used in phylogenetic analysis, it can often yield high support values despite abundant incongruence between gene trees (Minh *et al.*, 2020*a*). Thus, the site concordance factors (sCF) were calculated using the new algorithms first implemented in IQ-Tree v.2.2.2 to improve accuracy (Mo *et al.*, 2023) and were used to quantify potential discordance, because sCF estimates the concordance level of individual sites (Minh *et al.*, 2020*b*). The sCF were estimated using both a concatenated tree with 1000 ultrafast bootstraps and an edge-linked fully partitioned model and using 289 single-locus gene trees following the methodology outlined by The Lanfear Lab (http://www.robertlanfear.com/blog/files/concordance_factors.html).

#### Coalescent-based species trees

Coalescent species trees were inferred using ASTRAL III 5.7.8 (Zhang *et al.*, 2018), by inputting the generated gene tree files that were based on the alignments of each gene. ASTRAL was used to estimate quartet support (-t 1) for the recovered topology at each node of the tree. Then the conflict, concordance and phylogenetic signal were estimated using the PhyParts script (Smith *et al.*, 2015) and visualized using the PhyParts PieCharts script (https://github.com/mossmatters/MJPythonNotebooks/blob/master/PhyParts_PieCharts.ipynb). The PhyParts PieCharts script infers the number of gene trees that support, oppose or provide no information on a particular node with respect to the dominant species tree topology and maps these values onto the tree in a pie chart format.

## RESULTS

### Sequencing, quality control and target capture

**Fig. 1.**
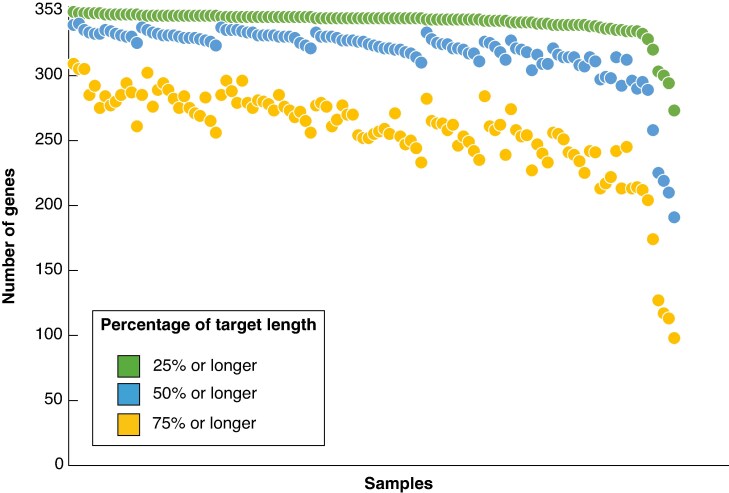
The number of genes recovered per sample was categorized by the percentage of the target length recovered (green = >25 %, blue = >50 % and yellow = >75 %).

The Illumina NovaSeq 6000 sequencing resulted in reads ranging from 408 021 to 9 596 685 with a mean of 4 678 487 per sample (Table 1). After initial quality control and trimming in the HybPiper-nf pipeline, the total number of paired-end reads ranged from 381 554 to 8 888 664, with a mean of 4 367 361. The mapping of reads to targets in HybPiper was variable and ranged from 3 % (98 868 reads) to 24.9 % (2 508 354 reads) with a mean of 10.07 % (832 533 reads) (Table 1). Read mapping to targets recovered a total of 353 genes (100 %), with 344 being the lowest number of genes recovered per sample, from the 35 000 targets (353 genes), with sequences that were >25 % of the target length ranging from 273 to 349 genes with a mean of 341. Sequences that were >50 % of the target length ranged from 191 to 340 genes with a mean of 318, and sequences at >75 % of the target length ranged from 98 to 309 genes with a mean of 257 (Fig. 1; Table 1), which is exceptional gene recovery (for complete outputs from HybPiper, see Supplementary Data Table S2). Data are available from the SRA (project number PRJNA1270708).

**Table 1.** Read filtering and assembly results from HybPiper.

	No. raw reads (paired)	No. trimmed reads (paired)	No. reads mapped to target	No. genes recovered	% target length >25 %	% target length >50 %	% target length >75 %	No. paralogue warnings
Minimum	408 021	720 166	98 868 (3 %)	344	273	191	98	0
Mean	4 678 487.112	4 367 361.159	1 877 200.765 (22 %)	351	341	318	257	4
Median	4 605 167	4 269 683	832 533 (10.7 %)	351	344	325	262	4
Maximum	9 596 685	17 516 768	2 508 354 (24.9 %)	353	349	340	309	19
Total	–	955 336 164	108 603 450 (11.06 %)	–	–	–	–	–

No. raw reads (paired) = numbers of RAW paired-end Illumina reads before filtering; No. reads mapped to target = number of reads mapped to baits target sequences; No. genes recovered = number of genes recovered per sample; % target length >25 % = sequences that were >25 % of the target length or greater; % target length >50 % = sequences that were >50 % of the target length or greater; % target length >75 % = sequences that were >25 % of the target length or greater; No. paralogue warnings = number of genes flagged with paralogue warnings.

### Marker selection for phylogenetic inference

From the 353 genes recovered from HybPiper pipeline, the paragone-nf pipeline was used to remove paralogous genes, which resulted in a final set of 299 genes, after filtering, to be used for downstream analysis, with each final gene alignment ranging from 93 to 3302 bp in length (Table 1). Each alignment was visually inspected using Geneious, and 289 genes, which ranged from 103 to 3302 bp in length, were selected based on alignment quality and inclusion of >80 % of species within the alignment. The concatenation of individual gene alignments in the resulting partitioned alignment had 33.3–85.6 % variable sites and 11.4–54.6 % parsimony-informative sites (Table 2).

**Table 2.** Phylogenetic tree statistics generated using IQ-Tree.

Type	Mean	Minimum	Maximum
Number of taxa	113.66	68	115
Percentage of taxa	98.83	59.1	100
Alignment length	725.6	103	3660
Percentage of variable sites	58.16	33.33	85.55
Percentage of parsimony-informative sites	30.24	11.42	54.57

The table shows the total number of taxa in each partition of the matrix, the total percentage of taxa included in the matrix, the length of the alignment for each partition, the percentage of variable sites in each partition and the percentage of sites that were parsimony informative in each partition.

### Phylogenetic reconstruction of Mirbelieae

#### Maximum likelihood

**Fig. 2.**
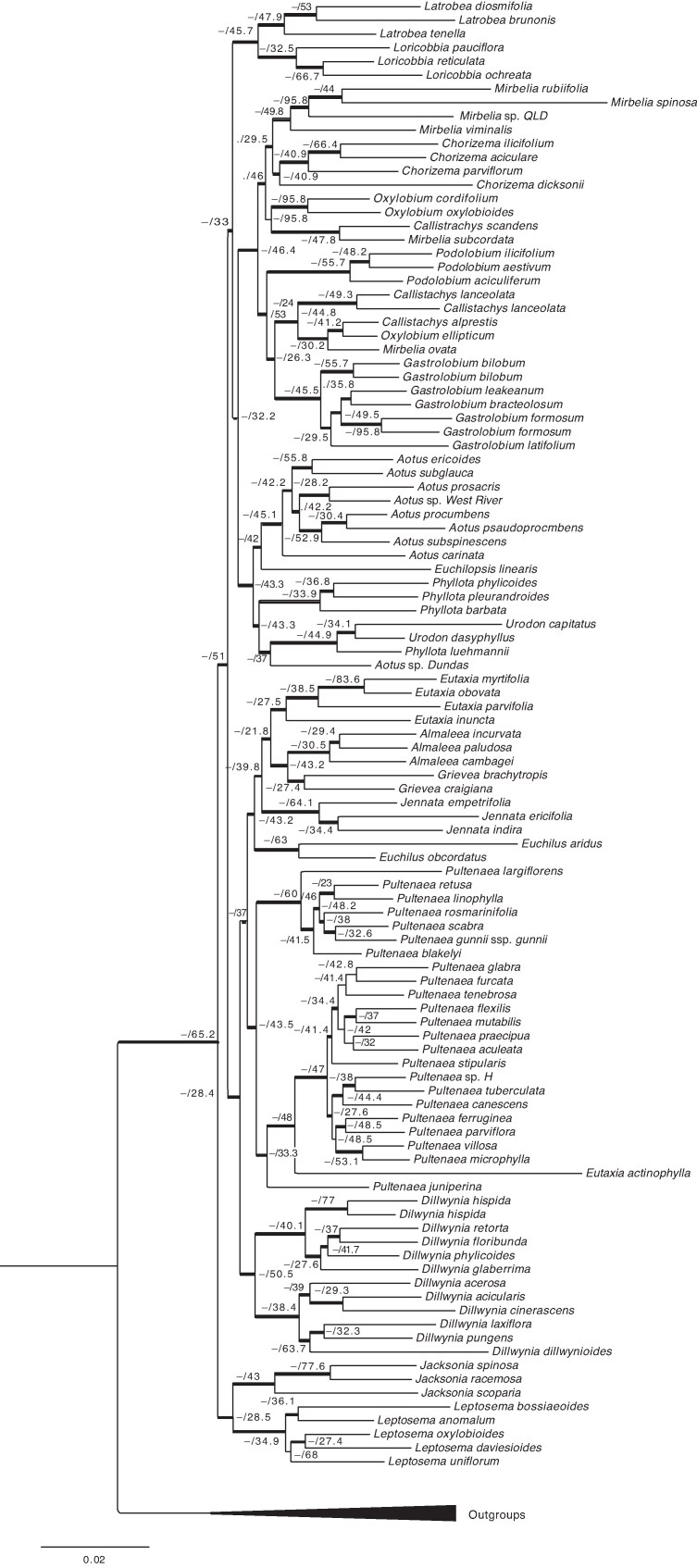
Maximum likelihood phylogenetic tree showing 115 taxa in Mirbelieae, including outgroups. The numbers at each node indicate the following: (1) SH-like approximate likelihood ratio test (SH-aLRT); (2) UltraFast Bootstrap (UFBoot); and (3) site concordance factors (sCF). The SH-aLRT or/and UFBoot values of >95 % are indicated by a thickened branch and -/ at each node.

**Fig. 3.**
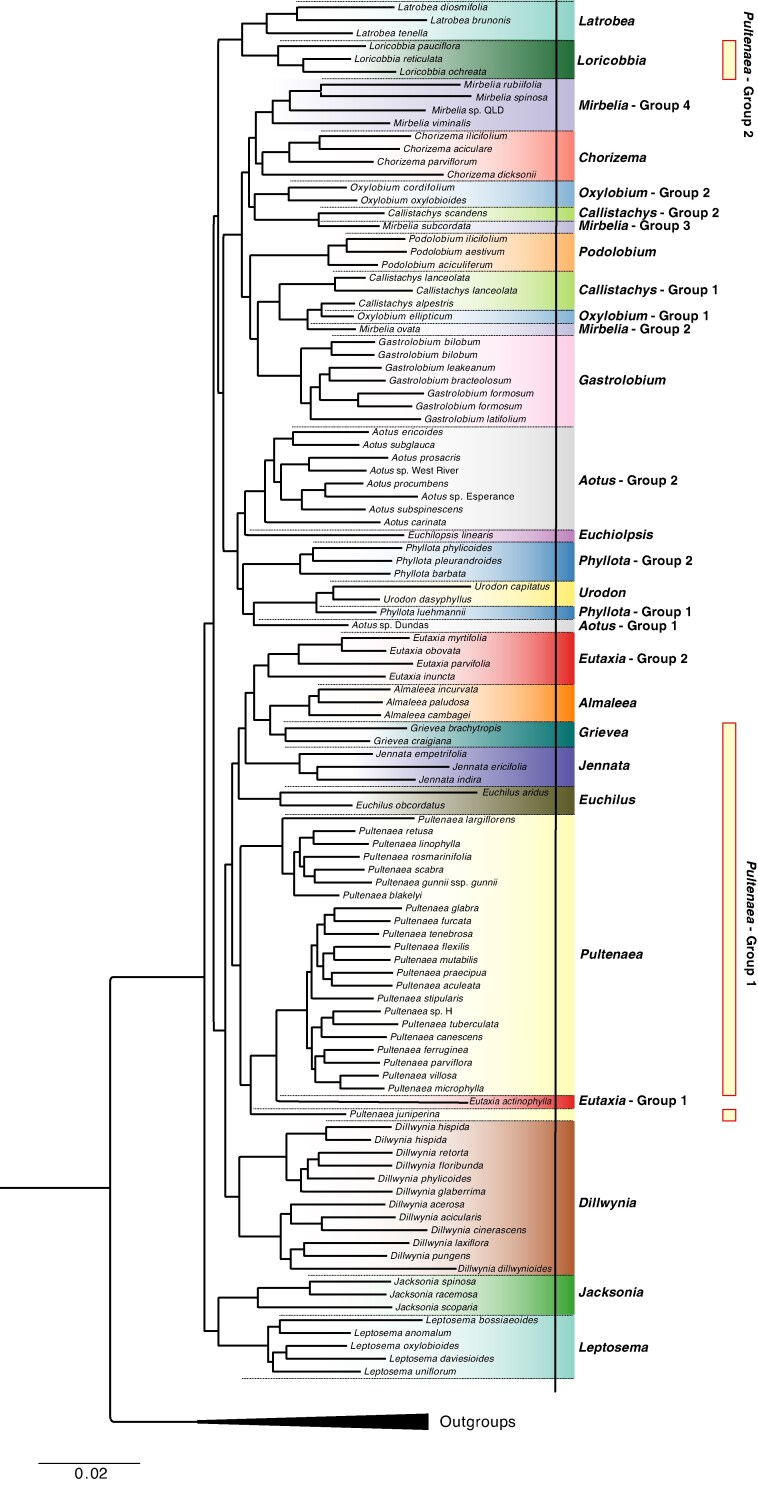
Maximum likelihood phylogenetic tree of Mirbelieae with generic groupings. Each separate colour represents a genus based on the current taxonomy (*sensu* Barrett *et al.*, 2021, 2024*c*). Each genus with more than a single group is separated, e.g. *Aotus* Group 1 and *Aotus* Group 2. The bounded boxes reflect recent taxonomic changes in *Pultenaea s.l.* to the right of the figure, indicating the Crisp and Weston (1995) concept of *Pultenaea* and two groups of *Pultenaea s.l.* recovered.

The reconstructed concatenated maximum likelihood phylogenetic tree generated using IQ-Tree and 289 markers/genes (209 271 bases), showed overall good resolution, with all nodes returning bootstrap values of >95 % or higher and sCF ≥ 21.8 % (Fig. 2). Of the 17 traditionally recognized genera included in this study, 11 were found to be monophyletic, with bootstrap support ≥ 95 %: *Almaleea* Crisp & P.H.Weston, *Chorizema* Labill., *Dillwynia* Sm., *Euchilopsis* F.Muell., *Eutaxia* R.Br., *Gastrolobium*, *Jacksonia* R.Br. ex Sm., *Latrobea* Meisn., *Leptosema* Benth., *Podolobium* R.Br. and *Urodon* Turcz.). However, *Aotus* Sm., *Callistachys* Vent., *Mirbelia* Sm., *Oxylobium* Andrews, *Phyllota* (DC.) DC. ex Benth. and *Pultenaea sensu* Crisp and Weston (1995) were found to be polyphyletic (Fig. 3). The most densely sampled genus, *Pultenaea sensu* Crisp and Weston (1995), represented by 33 samples, has representatives in two major clades. The first group comprises 30 taxa from varying geographical ranges, including species that were recently transferred to the new genera *Grievea* R.L.Barrett, Clugston & Orthia and *Jennata* R.L.Barrett, Clugston & Orthia, and the reinstated *Euchilus* R.Br. by Barrett *et al.* (2024*a*, *b*). The group is monophyletic only if *Eutaxia* and *Almaleea* are included, rendering the first group of *Pultenaea sensu* Crisp and Weston (1995) paraphyletic. The second group is small, consisting of a clade of taxa from Western Australia, which Barrett et al. (2024*a*, *b*) transferred to the new genus *Loricobbia* R.L.Barrett, Clugston & Orthia [*L. pauciflora* (M.B.Scott) R.L.Barrett & T.D.Macfarl., *L. reticulata* (Sm.) R.L.Barrett & T.D.Macfarl. and *L. ochreata* (Meisn.) R.L.Barrett & T.D.Macfarl.], which are sister to the sympatric Western Australian genus *Latrobea*.

#### Coalescent-based species tree

The coalescent-based species tree produced in ASTRAL used the same 289 markers and resulted in a tree topology (Supplementary Data Fig. S1) that was highly congruent with the maximum likelihood-based tree (Fig. 2). Of 106 nodes in each of these two trees, 96 are congruent, including 9 of the 10 most basal nodes in the maximum likelihood tree. The coalescent-based species tree showed some gene tree incongruence, indicated by low quartet support (1–192) values generated by ASTRAL, especially in the most basal nodes (Supplementary Data Fig. S1).

#### Gene and site concordance

Site concordance factors were mapped on the maximum likelihood-based gene tree (Fig. 2) and ranged from 21.8 to 95.8 % with a mean of 44.62 %, indicating good site concordance support for the reference topology from the gene trees relative to the number of informative sites supporting the grouping of a given node. The sCF were consistent with the quartet support values provided by ASTRAL.

## DISCUSSION

The myBaits Angiosperms353 universal baits set has already shown great promise for its use in both the PAFToL and GAP projects, which are large-scale phylogenomics projects that have investigated the phylogenies of angiosperm genera (Baker *et al.*, 2022; Zuntini *et al.*, 2024; Simpson *et al.*, 2025). For Mirbelieae, the Angiosperms353 bait set showed remarkable marker/gene recovery, with the maximum number of 353 genes being recovered across our data set (100 % of target genes; Fig. 1) and a mean of 351 genes recovered per sample, and 289 genes (81.9 %) were found to be free of paralogues and included in the final assembly, after filtering and quality control.

When these results are compared with other studies using the Angiosperms353 bait set, higher numbers of genes were recovered for Mirbelieae (Fabaceae) than for other plant groups (Fig. 1). For example, the analysis of the family Cornaceae recovered a total of 348 genes with a mean of 325 (Thomas *et al.*, 2021), whereas Clarkson *et al.* (2021) recovered 353 genes with a mean of 335 in Apiaceae, and Chincoya and Solórzano (2022) recovered only 319 genes with a mean of 173 in the Caryophyllids. This indicates that for the tribe Mirbelieae, our results yielded a higher-than-average ratio of gene recovery (Johnson *et al.*, 2019), and our resulting phylogenetic trees resolve the deep nodes of the phylogeny, with all major clades being supported by >95 % (UFBoot; Fig. 2), thereby demonstrating the potential utility of the Angiosperms353 bait set more broadly in legume systematics.

### Resolving phylogenetic relationships in tribe Mirbelieae

Our analyses of sequences targeted by the Angiosperms353 universal baits set largely resolved the relationships between major clades in Mirbelieae (Fabaceae). The tree derived by maximum likelihood analysis of concatenated data was highly congruent with that produced by the coalescent-based analysis of ASTRAL (Figs 1 and 2). These trees were broadly congruent with the results of earlier studies that found several genera to be paraphyletic as then circumscribed, based on Sanger-generated sequence data, although these studies lacked bootstrap support for most major clades (Orthia *et al.*, 2005*a*, *b*; Barrett *et al.*, 2021). However, for the first time, we have produced a phylogenetic tree with support for most currently recognized genera. A durable genus-level classification for the tribe can now be proposed for several clades that formed polytomies in previous studies.

The topologies of our phylogenetic trees (Figs 1 and 2) show that 11 of the 17 sampled genera (108 taxa excluding outgroup taxa) in the Mirbelieae were monophyletic, including *Almaleea* (3 species), *Chorizema* (4), *Dillwynia* (12), *Euchilopsis* (1), *Eutaxia* (5), *Gastrolobium* (8), *Jacksonia* (3), *Latrobe* (3), *Leptosema* (5), *Podolobium* (5) and *Urodon* (2) (Fig. 3). On the contrary, *Aotus* (9), *Callistachys* (3), *Mirbelia* (8), *Oxylobium* (3), *Phyllota* (5) and *Pultenaea sensu* Crisp and Weston (1995) (32) were found to be polyphyletic or paraphyletic. Both *Eutaxia* and *Almaleea* sit within *Pultenaea* Group 1 (Fig. 3), highlighting the problems associated with the generic classification of *Pultenaea sensu* Crisp and Weston (1995). Given the size of Fabaceae, challenges to circumscription of genera based on morphological data are nothing new, and the ‘resolution of relationships with molecular data is crucial for progress’ (LPWG, 2017).

Although the branch length of *Mirbeilia diliata* is above average, high data quality scores suggest that this extended branch length is likely to reflects the geographical isolation of this morphologically unique Western Australian species from its nearest relatives in eastern Australia. Its phylogenetic placement aligns with expectations based on morphology. The position of *Eutaxia actinophylla* Chappell & C.F.Wilkins is anomalous in comparison to other *Eutaxa* species included in our study. Although marker recovery exceeded 80 % and sequencing alignment quality fell within acceptable parameters, the corresponding long branch lengths of the taxa raise concerns regarding their position in the presented phylogenies (Figs 2, 3). However, owing to limited sampling within the tribe, we are hesitant to discard the sample. The position of *Pultenaea juniperina*, on a long branch sister to *E. actinophylla* and a large clade of *Pultenaea* (Figs 2, 3), would be likely to improve with the addition of more close relatives of *P. juniperina*, which are currently lacking in our sampling of the genus. We acknowledge that the isolation of this sample might be causing long-branch attraction to the *Eutaxia actinophylla* sample, and further sampling is required to confirm or reject the monophyly of *Eutaxia*.

When comparing the topology of our phylogeny (Fig. 2) with earlier studies, neither Crisp and Weston (1987, 1995) nor Crisp and Cook (2003) nor Orthia *et al.* (2005*a*, *b*) were able to resolve relationships among many genera within the tribe. This raised concerns within the botanical community that relationships within the tribe might be irresolvable owing to rapid radiation or other evolutionary processes (Orthia *et al.*, 2005*a*, *b*). The most comprehensively sampled phylogeny of core Mirbelieae, which included 200 taxa and all currently recognized genera, was produced by Barrett *et al.* (2021). However, they used only a single plastid marker (*trn*L-F), which provided little statistical support for relationships between these genera. Nonetheless, heir results indicated that the largest genus in Mirbelieae, *Pultenaea*, was polyphyletic, a consistent result in all molecular studies to date.

### High bootstrap support values and larger marker datasets

Our resulting phylogenetic tree (Fig. 2) shows good branch support (based on ultrafast bootstrap support). However, in phylogenomic datasets incorporating many markers, high branch support can often be misleading because branch support values, such as bootstrap or posterior probability, are unable to detect discordance between gene trees and species trees (Roycroft *et al.*, 2019; Chan *et al.*, 2020). Discordance between genes can be attributable to many processes, including homoplasy within gene trees, hybridization, gene and genome duplication, horizontal gene transfer and incomplete lineage sorting (ILS) (Léveillé-Bourret *et al.*, 2018).

The use of Angiosperms353 provided strong support for our maximum likelihood phylogenetic tree (Fig. 2), with sCF indicating medium to high support (mean = 44.62 %); the ultrafast bootstrap shows support across most nodes, with 95 % or greater support for all nodes of the tree. Our results compare well with those of other studies based on the Angiosperms353 bait set. For example, Giaretta *et al.* (2022) used Angiosperms353 to study the genus *Eugenia* L. (Myrtaceae) and found bootstrap-supported deep nodes, with average sCF (65.4 %) being similar to ours. Using sCF is important because traditional bootstrap may not measure branch support accurately on larger marker set phylogenies (Chan *et al.*, 2020). Our results for the Mirbelieae show that although the sCF is lower than our bootstrap support, sCF could still provide strong support for the tree topology (Fig. 2).

When we compare the maximum likelihood phylogenetic tree (Fig. 2) with the coalescent phylogenetic tree generated in ASTRAL (Supplementary Data Fig. S1), their topologies are largely congruent. However, the concordance factors calculated by ASTRAL are lower than those calculated through IQ-Tree, suggesting some gene conflict from ASTRAL or potentially differential algorithms for calculating support (see Zhang *et al.*, 2018). Although this could indicate that incomplete lineage sorting, hybridization or rapid radiation around the deeper nodes might be playing a role in gene tree conflict in ASTRAL (Reddy *et al.*, 2017), the maximum likelihood tree was mostly unaffected (see discussion by Walker *et al.*, 2017; Carlsen *et al.*, 2018; Stull *et al.*, 2021; Carruthers *et al.*, 2022). Carlsen *et al.* (2018) found that gene and genome duplication could directly influence gene conflict in the phylogenetic signal, indicating that rapid radiation can result in gene conflict in phylogenies. However, despite our high marker recovery, few paralogues were detected, indicating that gene duplication is not likely to have been a confounding process in Mirbelieae.

The 289 markers used from Angiosperm353 are generally conserved (McDonnell et al., 2021); the markers provided sufficient phylogenetic signal to resolve phylogeny, despite the possibility that Mirbelieae has undergone rapid evolution. The discrepancies in support between our maximum likelihood and coalescence phylogenetic trees (Figs 2 and 3) are likely to be attributable to the differences in the mathematical models selected for the support indices that each method can use. Additionally, the sCF from IQ-Tree was calculated using the new algorithms first introduced in IQ-Tree v.2.2.2, aiming to enhance accuracy in the larger and denser marker set datasets (Mo *et al.*, 2023), which accounts for our strong sCF. Overall, our findings demonstrate robust bootstrap support (Fig. 2) and indicate ample phylogenetic signal in the data to provide valuable insights into relationships within Mirbelieae. This enables us to deduce generic relationships among genera within the group and establishes a solid groundwork for a more detailed investigation into the origins and diversification within the tribe.

### Phylogenetic relationships of taxa within Mirbelieae

Although our resulting trees included only ∼20 % of taxa in Mirbelieae, our sampling was undertaken carefully to ensure representation of morphological and biogeographical diversity in the tribe. Significantly, *Almaleea*, *Chorizema*, *Dillwynia*, *Euchilopsis*, *Eutaxia*, *Gastrolobium*, *Jacksonia*, *Latrobea*, *Leptosema*, *Podolobium* and *Urodon* were found to be monophyletic and supported as discrete genera (Figs 2, 3). These results demonstrate that the phylogenetic relationships of monophyletic taxa are directly correlated with traditional morphology and taxonomy to a high degree (Barrett *et al.*, 2021, 2024*c*).

However, *Aotus*, *Callistachys*, *Mirbelia*, *Oxylobium*, *Phyllota* and *Pultenaea sensu* Crisp and Weston (1995) were found to be either polyphyletic or paraphyletic (Figs 2, 3), which indicates homoplasy amongst the morphological characters traditionally used to define these genera. This highlights the importance of careful sampling, which is crucial to improving generic classification within Mirbelieae. Our findings contrast with those of Barrett *et al.* (2021), because we found *Mirbelia* and *Phyllota* to be polyphyletic, possibly owing to differences in taxon sampling (200 versus 115 in this study) or owing to the use of a single genetic marker when compared with 289 in this study. Our results indicate that the morphological characters used to define genera within Mirbelieae are not always consistent with their phylogenetic relationships.

*Pultenaea sensu* Crisp and Weston (1995) is the largest genus in Mirbelieae, and our results showed that species fell into two main clades within the broader Mirbelieae (Fig. 2): the core *Pultenaea* (Group 1; with species in eastern and Western Australia) and a small group of species endemic to Western Australia (Group 2; Orthia *et al.*, 2005*c*). Species in Group 1 do not form a monophyletic group, because *Eutaxia* and *Almaleea* are also nested within this clade, and both are defined by distinct morphology (Crisp and Weston, 1991; Wilkins *et al.*, 2010). The morphological distinction of several clades had already been recognized by Orthia *et al.* (2005*c*), and their *Pultenaea* clades D and E (the *P. quaerita* and *P. ericifolia* groups) correspond to the clades we recovered as being more closely related to *Almaleea* and *Eutaxia* rather than their *Pultenaea* clades A–C. Their clade F (the *P. reticulata* group) was likewise recovered as sister to *Latrobea*.

To rectify the supported polyphyly of *Pultenaea* reported here, Barrett *et al.* (2024*c*) reinstated the genus *Euchilus* and created three new genera (*Grievea*, *Jennata* and *Loricobbia*), thereby creating a monophyletic concept of *Pultenaea*. These taxonomic changes were supported by our present results and are strongly congruent with diagnostic morphological features (Orthia *et al.*, 2005*c*, Barrett *et al.*, 2024*c*: tables 1 and 2). Congruent with earlier studies based on Sanger sequence data, Zuntini *et al.* (2024) have confirmed that the monotypic genus *Stonesiella* Crisp & P.H.Weston also belongs in this clade.

This combined *Pultenaea* clade 1, plus *Almaleea*, *Eutaxia* and *Stonesiella*, can now be divided into seven subclades. All seven subclades can be recognized readily using morphological characters, and therefore we believe they can be classified readily at generic rank.

1. *Pulteanea sensu stricto*, with all but one species (*Pultenaea tenuifolia* R.Br. ex Sims) endemic to eastern Australia.
2. The reinstated genus *Euchilus* (formerly the *Pultenaea quaerita* group), with all but one species endemic to Western Australia.
3. The recently named genus *Jennata* (formerly the *Pultenaea ericifolia* group), endemic to Western Australia.
4. The recently named genus *Grievea* (formerly the *Pultenaea brachytropis*–*P. craigiana* group), endemic to Western Australia.
5. *Stonesiella*, endemic to eastern Australia.
6. *Almaleea*, endemic to eastern Australia.
7. *Eutaxia*, with species in both eastern and Western Australia.

Recognition of *Euchilus*, *Grievea* and *Jennata* required the least number of name changes to create monophyletic generic concepts in this clade, in part because many species in the *Euchilus* clade already had names available in that genus. The three species of *Almaleea* form a clade that is sister to a small clade consisting of *Pultenaea brachytropis* Benth. and *Pultenaea craigiana* C.F.Wilkins, Orthia & Crisp, and, in turn, they are all sister to the four species of the monophyletic *Eutaxia* (see Wilkins *et al.*, 2009). Morphological examination suggests that *P*. *brachytropis* and *P. craigiana* are dissimilar to *Almaleea*, justifying their recognition as a distinct genus, *Grievea*. We acknowledge that *Pultenaea adunca* Turcz. also belongs in this clade (Barrett *et al.*, 2021), but its relationships to these genera remain uncertain.

*Aotus* is split into two main groups: Group 1 contains only *Aotus* sp. Dundas (M.A.Burgman 2835), and Group 2 with nine other species, which is sister to *Euchilopsis*. The species in Group 2 are monophyletic and can together be recognized by morphological characters. *Phyllota* is separated into two groups, with the inclusion of *Aotus* sp. Dundas and two *Urodon* species in the same clade. The definition of genera in this group has been problematic for some time, with Crisp and Weston (1995) proposing that an additional genus might be needed. An alternative option might be the expansion of *Urodon* to include *Phyllota luehmannii* F.Muell. and *Aotus* sp. Dundas, both of which have morphological affinities to *Urodon*. An expanded *Urodon* would form a sister genus to *Phyllota s.s*. Both *Oxylobium* and *Callistachys* are polyphyletic (Fig. 2), but denser sampling is required to infer their detailed phylogenetic relationships.

### Generic limits in Mirbelieae

Taking the recent reclassification of *Pultenaea* into account (Barrett *et al.*, 2024*c*), our results support the ongoing recognition of 16 monophyletic genera (*Almaleea*, *Chorizema*, *Dillwynia*, *Euchilopsis*, *Euchilus*, *Eutaxia*, *Gastrolobium*, *Grievea*, *Jacksonia*, *Jennata*, *Latrobea*, *Leptosema*, *Loricobbia*, *Podolobium*, *Pultenaea* and *Urodon*), because this presents the greatest taxonomic and nomenclatural stability, and there are now established morphological concepts for each of these genera. *Mirbelia*, *Oxylobium*, *Phyllota*, *Podolobium* and *Urodon* are all likely to require some minor modification to their circumscriptions.

For the genera that were shown to be polyphyletic (*Aotus*, *Callistachys*, *Eutaxia*, *Mirbelia*, *Oxylobium* and *Phyllota*; Fig. 3), each case must be considered based on phylogenetic relationships, morphological distinctiveness and nomenclatural stability. Further sampling is likely to be required for some clades before new classifications can be proposed. *Aotus* is paraphyletic, because a single taxon, *Aotus* sp. Dundas, is more closely related to *Urodon*, a position previously suspected based on morphological affinity. The main *Aotus* clade recovered here contains eight taxa (44 % of accepted species), and it appears that the genus can be rendered monophyletic simply by the removal of the informally named *Aotus* sp. Dundas. *Phyllota* is represented by 5 of the 10 currently accepted species (50 %), and although four of the five taxa group together, *Phyllota luehmannii* sits as sister to *Urodon*. This species was somewhat anomalous in *Phyllota*, hence a close relationship with *Urodon* is not surprising, and the two genera are closely related in any case.

Barrett *et al.* (2021) recovered a tree in which *Mirbelia* was monophyletic based on the samples included, but as our results show, a small number of additional species do not resolve within the main *Mirbelia* clade. This suggests that the morphological character that currently defines *Mirbelia* (a dividing septum in the fruit) requires reassessment. With only 7 taxa represented of 25 accepted species (28 %), more detailed sampling, including all taxa of *Callistachys* (*sensu* Barrett *et al.*, 2021) and *Oxylobium*, is required prior to any re-circumscription of *Mirbelia*. The position of *Podolobium* (*Callistachys*) *scandens* is conflicting between Barrett *et al.* (2021) and our results, and it is possible that one of these results is erroneous owing to sample contamination.

*Pultenaea* is the largest and most diverse genus in Mirbelieae, containing >130 named species (Orthia *et al.*, 2005*a*, *b*, *c*; Renner *et al.*, 2022; Telford *et al.*, 2022; Barrett *et al.*, 2024*a*, *b*), and our assembly includes only 25.5 % of these (33 species). As outlined above, *Pultenaea*, as it is traditionally defined, needed to be split into five separate genera to achieve monophyly. A small group represented by three taxa, all endemic to south-west Western Australia, is sister to *Latrobea* and only distantly related to *Pultenaea s.s*. This clade is also morphologically distinct from *Latrobea*, hence it has been named a new genus, *Loricobbia*. Our results show that the majority of species in the traditional concept of *Pultenaea* form a single clade that also includes *Almaleea* and *Eutaxia* (based on Zuntini *et al.*, 2014; *Stonesiella*). Most of the species traditionally included in *Pultenaea* form a clade (based on our limited sampling), and all but one of these species is endemic to eastern Australia, providing clear geographical cohesion. This core *Pultenaea* clade is also readily definable morphologically (Barrett *et al.*, 2024*c*). An expanded *Pultenaea* would have contained significant morphological variability, and a synapomorphy for the broader clade 1 (*Pultenaea s.l.* plus *Almaleea*, *Eutaxia* and *Stonesiella*) is yet to be identified. Orthia *et al.* (2005*b*) suggested that one way to resolve the genetic limits in Mirbelieae would have been to combine all core Mirbelioid genera into a greatly expanded *Pultenaea*, but this option left much to be desired, because 76 % of taxa in that clade would have changed names, and the resulting mega-genus would have been highly morphologically diverse and difficult to define. Conversely, establishing a small number of additional genera or modest modifications to existing genera will require <8 % of names to change to establish monophyletic genera (Barrett *et al.*, 2021, 2024*c*), and we are progressively enacting this as the preferable option.

In addition to changes still required at generic rank in Mirbelieae, species diversity in the already large genus *Pultenaea* remains significantly under-described, with current morphology-based species definitions appearing to be too broad in many instances. This has resulted in multiple species groups being recognized as highly variable morphological entities. For example, Renner *et al.* (2022) used population genomics to assess genetic differences between different morphotypes of *Pultenaea glabra* Benth. Their work demonstrated that *P. glabra* represented a species complex composed of nine genetically and morphologically distinct entities, with eight being new to science. Likewise, Barrett *et al.* (2024*a*) found support for 18 species (14 requiring new names) in the *Pultenaea setulosa* species complex, which could indicate that there are as many if not more undescribed species in other species complexes, e.g. *P. polifolia* and *P. procumbens s.l*. Notably, Renner *et al.* (2022) also found evidence for hybridization between *Pultenaea flexilis* Sm. and *P. glabra*. Given this, hybridization can also be expected to occur in other Mirbelieae genera.

To assess putative hybridization in target capture datasets properly, both parental species are needed to determine the hybrid status of an individual (Nauheimer *et al.*, 2021). These results might help to explain incidences of minor incongruence between gene trees and species trees (Fig. 2; Supplementary Data Fig. S1) in the present study (see Twyford and Ennos, 2012). Our current sampling suggests that *Pultenaea s.s.* has three major clades. If more detailed sampling supports these clades, then it might be justified to recognize them as subgenera to facilitate our understanding of diversity within this large genus.

### Conclusion

Core genera in Mirbelieae (Fabaceae/Leguminosae) represent a significant proportion of Australia’s pea-flowered legume diversity (32 %), but until now their phylogenetic relationships have been largely unresolved. However, advances in next generation sequencing and targeted baits capture are finally enabling us to reveal previously obscure relationships. Universal approaches, such as the Angiosperms353 targeted baits sets, are providing unprecedented resolution to resolve phylogenetic relationships within angiosperms (Zuntini *et al.*, 2024).

The paraphyly of *Pultenaea sensu* Crisp and Weston (1995) has been well demonstrated and is confirmed by our study. However, in contrast to previous studies, we present the first resolved phylogeny of Mirbelieae and demonstrate that 11 of the 17 traditionally recognized genera in core Mirbelieae are monophyletic. With confidence that relationships between *Pultenaea* and other genera of core Mirbelieae are supported, a new genus-level classification is now both possible and justified for major clades. Specifically, these results have been used to create a monophyletic concept of *Pultenaea*, the largest genus in the tribe, through the reinstatement of *Euchilus* and the description of three new genera (Barrett *et al.*, 2024*c*).

The Angiosperms353 universal bait set has proved to be of great value for legume phylogenetics and understanding plant relationships. For phylogenomic data to inform the taxonomy of some small clades within Mirbelieae, greater taxon sampling is still required. Nevertheless, our results demonstrate that target enrichment sequencing for the generation of phylogenomic data can provide an excellent foundation for resolving and stabilizing taxonomic concepts and providing a new understanding of morphological diversity in the Mirbelieae.

## Supplementary Material

mcaf128_Supplementary_Data

## SUPPLEMENTARY DATA

Supplementary data are available at *Annals of Botany* online and consist of the following. Table S1: table showing all samples. Table S2: table showing the recovery statistics from the Hybpiper output for all samples. Figure S1: ASTRAL phylogenetic tree for tribe Mirbelieae. Node values above represent the number of markers concordant to a given node, and node values below indicate the number of discordant markers. Pie charts represent the proportion of markers that support the topology. Blue, markers supporting the topology; green, markers conflicting with the topology (most commonly a conflicting bipartition); red, markers conflicting with the topology (all other supported conflicting bipartitions); grey, markers with no support for a conflicting bipartition.
