# Supplementary material for: Resolving relationships in the radiation of Australia’s largest pea clade (Fabaceae tribe Mirbelieae) with target-capture sequencing
Source: Ann Bot. 2025 Jun 17;136(3):637–49. doi: 10.1093/aob/mcaf128 (PMC12455713; doi:10.1093/aob/mcaf128)
Supplement: mcaf128_Supplementary_Data [file mcaf128_supplementary_data.zip › Supplementary Figure 1.docx]

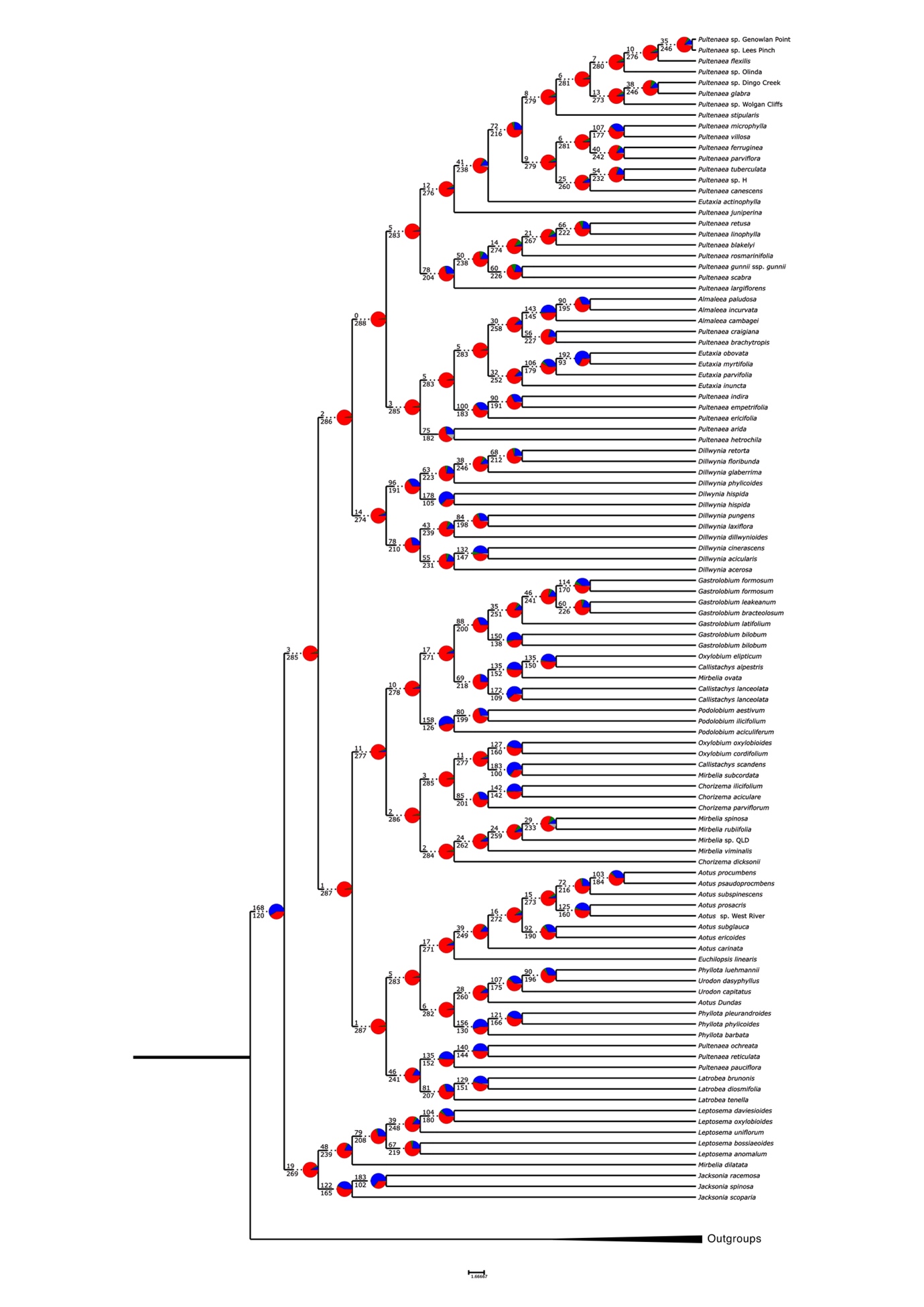


**Supplementary Figure 1**. ASTRAL phylogenetic tree for tribe Mirbelieae. Node values above represent the number of markers concordant to a given node, and node values below indicate the number of discordant markers. Pie charts represent the proportion of markers that support the topology. Blue: markers supporting the topology; Green: markers conflicting with the topology (most commonly a conflicting bipartition); Red: markers conflicting with the topology (all other supported conflicting bipartitions); Grey: markers with no support for a conflicting bipartition.
